# Crystal structure of (7-chloro-2-oxo-2*H*-chromen-4-yl)methyl *N*,*N*-di­methyl­carbamodi­thio­ate

**DOI:** 10.1107/S2056989015005678

**Published:** 2015-03-28

**Authors:** H. D. Kavitha, M. Vinduvahini, N. M. Mahabhaleshwaraiah, O. Kotresh, H. C. Devarajegowda

**Affiliations:** a Department of Physics, Govt. Science College, Hassan 573 201, Karnataka, India; b Department of Physics, Sri D Devaraja Urs Govt. First Grade College, Hunsur-571105, Mysore District, Karnataka, India; c Department of Chemistry, Karnatak University’s Karnatak Science College, Dharwad, Karnataka 580001, India; d Department of Chemistry, Karnatak Science College, Karnatak University, Dharwad, Karnataka 580001, India; e Department of Physics, Yuvaraja’s College (Constituent College), University of Mysore, Mysore 570 005, Karnataka, India

**Keywords:** crystal structure, 2*H*-chromene, C—H⋯S hydrogen bonds, π–π inter­actions

## Abstract

In the title compound, C_13_H_12_Cl N O_2_S_2_, the 2*H*-chromene ring system is almost planar, with a maximum deviation of 0.005 (2) Å. The packing features C—H⋯S hydrogen bonds and π–π inter­actions between fused benzene rings of chromene [shortest centroid–centroid distances = 3.6553 (13) and 3.5551 (13) Å].

## Related literature   

For biological applications of coumarins and di­thio­carbamates, see: Boas *et al.* (2004 ▸); D’hooghe & De Kimpe (2006 ▸); Fernández *et al.* (1995 ▸); Rao *et al.* (1981 ▸); Trkovnik *et al.* (1983 ▸). For a related structure and the synthesis, see: Mahabaleshwaraiah *et al.* (2012 ▸).
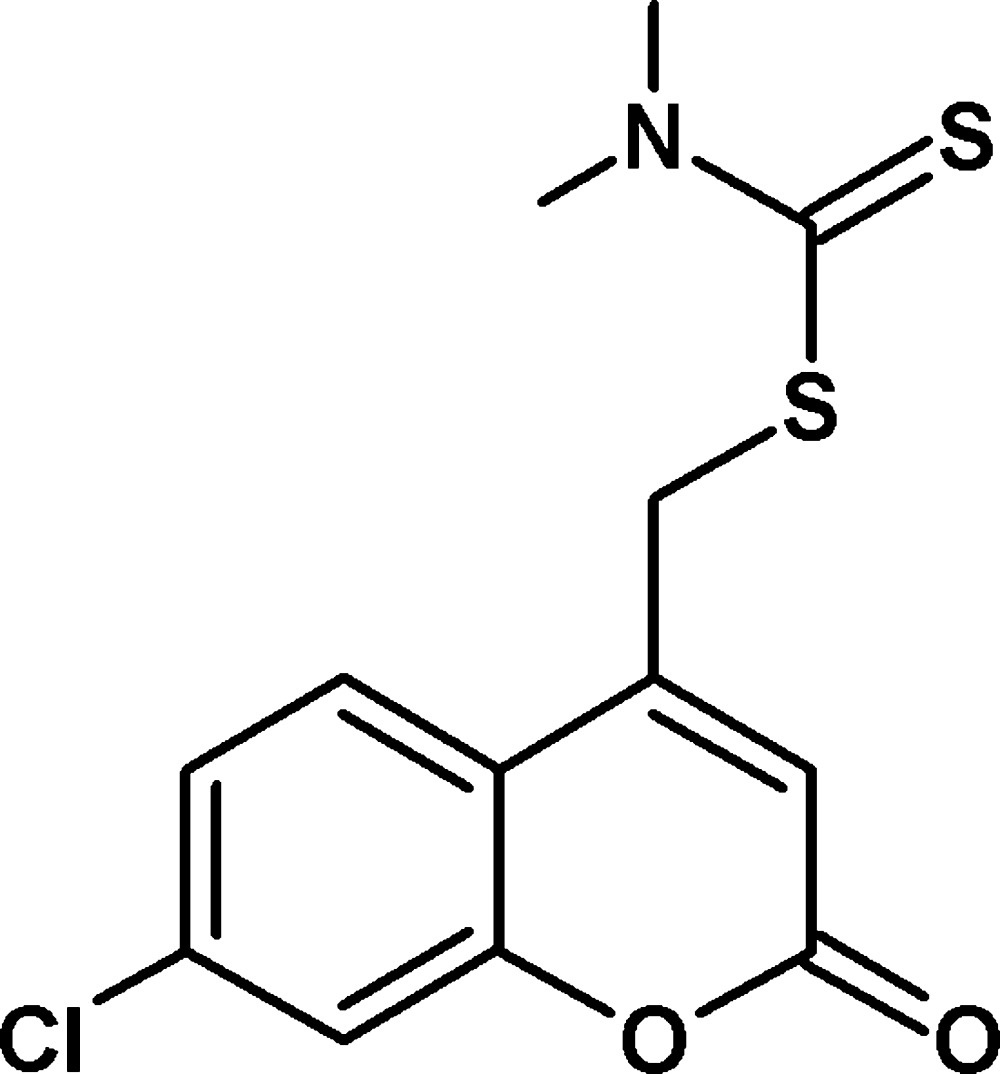


## Experimental   

### Crystal data  

C_13_H_12_ClNO_2_S_2_
*M* *_r_* = 313.81
Monoclinic, 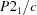

*a* = 9.7244 (4) Å
*b* = 7.1157 (3) Å
*c* = 20.0896 (9) Å
β = 94.404 (3)°
*V* = 1386.01 (10) Å^3^
*Z* = 4
Mo *K*α radiation
μ = 0.57 mm^−1^
*T* = 296 K
0.24 × 0.20 × 0.12 mm

### Data collection  

Bruker SMART CCD area-detector diffractometer
Absorption correction: ψ scan (*SADABS*; Sheldrick, 2007 ▸) *T*_min_ = 0.770, *T*_max_ = 1.000
16144 measured reflections
4752 independent reflections
2805 reflections with *I* > 2σ(*I*)
*R*_int_ = 0.029

### Refinement  

*R*[*F* ^2^ > 2σ(*F* ^2^)] = 0.053
*wR*(*F* ^2^) = 0.147
*S* = 1.03
4752 reflections
174 parameters
H-atom parameters constrained
Δρ_max_ = 0.32 e Å^−3^
Δρ_min_ = −0.35 e Å^−3^

### 

Data collection: *SMART* (Bruker, 2001 ▸); cell refinement: *SAINT* (Bruker, 2001 ▸); data reduction: *SAINT*; program(s) used to solve structure: *SHELXS97* (Sheldrick, 2008 ▸); program(s) used to refine structure: *SHELXL2014* (Sheldrick, 2015 ▸); molecular graphics: *ORTEP-3 for Windows* (Farrugia, 2012 ▸); software used to prepare material for publication: *SHELXL2014*.

## Supplementary Material

Crystal structure: contains datablock(s) I, global. DOI: 10.1107/S2056989015005678/bg2550sup1.cif

Structure factors: contains datablock(s) I. DOI: 10.1107/S2056989015005678/bg2550Isup2.hkl

Supporting information file. DOI: 10.1107/S2056989015005678/bg2550Isup3.cml

**Fig. 1.**
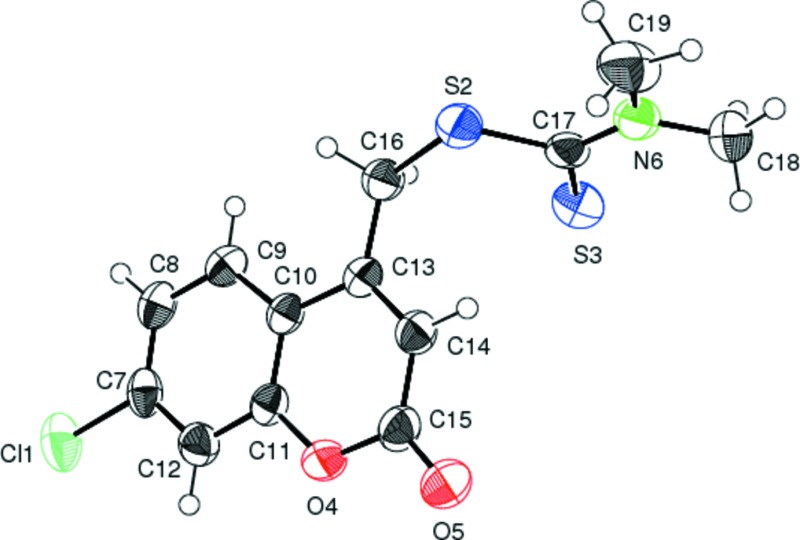
The molecular structure of the title compound. Displacement ellipsoids are drawn at the 50% probability level.

. DOI: 10.1107/S2056989015005678/bg2550fig1.tif
The mol­ecular structure of the title compound. Displacement ellipsoids are drawn at the 50% probability level.

**Fig. 2.**
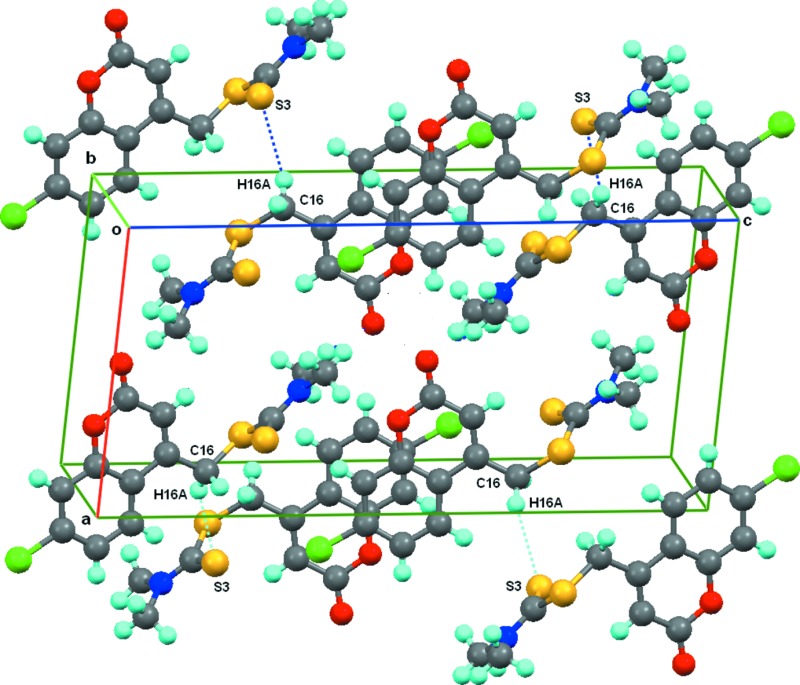
A packing view of the title compound. Dashed lines represent intermolecular interactions.

. DOI: 10.1107/S2056989015005678/bg2550fig2.tif
A packing view of the title compound. Dashed lines represent inter­molecular inter­actions.

CCDC reference: 1055112

Additional supporting information:  crystallographic information; 3D view; checkCIF report

## Figures and Tables

**Table 1:** Hydrogen-bond geometry (, )

*D*H*A*	*D*H	H*A*	*D* *A*	*D*H*A*
C16H16*A*S3^i^	0.97	2.84	3.707(2)	150

Symmetry code: (i) 
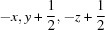
.

## supplementary crystallographic information

### S1. Comment

Synthetic coumarins are widely used as aroma chemicals because of their odour strength, tenacity, stability to alkali and relatively cheap price; applications include their use as a sweetener and fixative (in perfume); fragrance enhancers (for natural essential oils); blenders (in soaps and detergents); aroma enhancers (in tobacco); and for imparting pleasant odours to industrial products. The coumarins have been the subject of extensive studies because of their interesting biological activities and have, in fact, been used as therapeutic agents for the treatment of various diseases. Coumarins show quite diverse biological activities, including anticoagulant, anti-allergic, anthelmintic, diuretic, insecticidal and antibiotic properties (Trkovnik *et al.*, 1983); Rao *et al.*, 1981). The great electrophilicity of the nitrogen atom as compared to that of sulfur makes the latter more acidic and an active centre in the nucleophilic attack. The fact is that the sulfur anion formed is more stabilized by negative charge distribution. Furthermore, functionalized carbamates are an important class of compounds and their medicinal and biological properties warrant study (D'hooghe *et al.*, 2006). Organic dithiocarbamates are valuable synthetic intermediates (Boas *et al.*, 2004), which are ubiquitously found in a variety of biologically active compounds. Functionalization of the carbamate moiety offers an attractive method for the generation of derivatives, which may constitute interesting medicinal and biological properties (Fernández *et al.*, 1995).

The compound herein reported, (7-chloro-2-oxo-2*H*-chromen-4-yl)methyl dimethylcarbamodithioate (Fig. 1) presents a planar 2*H*-chromene ring system [maximum deviation: 0.005 (2) Å for atom C13]. The crystal structure shows intermolecular C—H···S bonds·(C16-H16A···S3, H···S= 2.84 Å; C-H···S: 150°) and π–π interactions between fused benzene rings of chromene [shortest centroid–centroid distances = 3.6553 (13) Å and 3.5551 (13) Å]. A packing view is shown in Fig. 2.

### S2. Experimental

All the chemicals used were of analytical reagent grade and were used directly without further purification. The title compound was synthesized according to the reported method (Mahabaleshwaraiah *et al.*, 2012). The compound is recrystallized by ethanol- chloroform mixture. Colourless needles of the title compound were grown from a mixed solution of Ethanol/Chloroform (V/V = 2/1) by slow evaporation at room temperature. Yield =72%; m.p.:405–407 K.

### S3. Refinement

All H atoms were positioned geometrically, with C—H = 0.93 Å for aromatic H, C—H = 0.97 Å for methylene H and C—H = 0.96 Å for methyl H, and refined using a riding model with *U*_iso_(H) = 1.5*U*_eq_(C) for methyl H and *U*_iso_(H) = 1.2*U*_eq_(C) for all other H.

### Crystal data

**Table:**

C_13_H_12_ClNO_2_S_2_	*D*_x_ = 1.504 Mg m^−^^3^
*M**_r_* = 313.81	Melting point: 407 K
Monoclinic, *P*2_1_/*c*	Mo *K*α radiation, λ = 0.71073 Å
*a* = 9.7244 (4) Å	Cell parameters from 2446 reflections
*b* = 7.1157 (3) Å	θ = 2.0–25.0°
*c* = 20.0896 (9) Å	µ = 0.57 mm^−^^1^
β = 94.404 (3)°	*T* = 296 K
*V* = 1386.01 (10) Å^3^	Plate, colourless
*Z* = 4	0.24 × 0.20 × 0.12 mm
*F*(000) = 648	

### Data collection

**Table:**

Bruker SMART CCD area-detector diffractometer	4752 independent reflections
Radiation source: fine-focus sealed tube	2805 reflections with *I* > 2σ(*I*)
Graphite monochromator	*R*_int_ = 0.029
ω and φ scans	θ_max_ = 32.2°, θ_min_ = 2.0°
Absorption correction: ψ scan (*SADABS*; Sheldrick, 2007)	*h* = −14→14
*T*_min_ = 0.770, *T*_max_ = 1.000	*k* = −10→10
16144 measured reflections	*l* = −27→30

### Refinement

**Table:**

Refinement on *F*^2^	Primary atom site location: structure-invariant direct methods
Least-squares matrix: full	Secondary atom site location: difference Fourier map
*R*[*F*^2^ > 2σ(*F*^2^)] = 0.053	Hydrogen site location: inferred from neighbouring sites
*wR*(*F*^2^) = 0.147	H-atom parameters constrained
*S* = 1.03	*w* = 1/[σ^2^(*F*_o_^2^) + (0.0636*P*)^2^ + 0.4178*P*] where *P* = (*F*_o_^2^ + 2*F*_c_^2^)/3
4752 reflections	(Δ/σ)_max_ = 0.043
174 parameters	Δρ_max_ = 0.32 e Å^−^^3^
0 restraints	Δρ_min_ = −0.35 e Å^−^^3^

### Special details

**Table:**

Experimental. IR (KBr, cm^-1^): 1722 (C=O), 1381 (C=S), 894(C—N). GCMS: m/e: 313; 1H NMR (400 MHz, CDCl_3_, \?, p.p.m): 3.38 (s, 3H, N—CH_3_), 3.47 (s, 3H, N—CH_3_), 4.80 (s, 2H, C4—CH_2_), 6.56 (s, 1H, C3—H), 7.45–7.92 (m, 3H, Ar—H). Mol. Formula: C_13_H_12_Cl N O_2_S_2_2; Elemental analysis: C, 49.75; H, 3.85; N, 4.46 (calculated); C, 49.67; H, 3.76; N, 4.39 (found).
Geometry. All e.s.d.'s (except the e.s.d. in the dihedral angle between two l.s. planes) are estimated using the full covariance matrix. The cell e.s.d.'s are taken into account individually in the estimation of e.s.d.'s in distances, angles and torsion angles; correlations between e.s.d.'s in cell parameters are only used when they are defined by crystal symmetry. An approximate (isotropic) treatment of cell e.s.d.'s is used for estimating e.s.d.'s involving l.s. planes.
Refinement. Refinement of *F*^2^ against ALL reflections. The weighted *R*-factor *wR* and goodness of fit *S* are based on *F*^2^, conventional *R*-factors *R* are based on *F*, with *F* set to zero for negative *F*^2^. The threshold expression of *F*^2^ > 2σ(*F*^2^) is used only for calculating *R*-factors(gt) *etc*. and is not relevant to the choice of reflections for refinement. *R*-factors based on *F*^2^ are statistically about twice as large as those based on *F*, and *R*- factors based on ALL data will be even larger.

### Fractional atomic coordinates and isotropic or equivalent isotropic displacement parameters (Å^2^)

**Table:**

	*x*	*y*	*z*	*U*_iso_*/*U*_eq_	
Cl1	−0.17680 (7)	0.68940 (10)	0.60871 (3)	0.0643 (2)	
S2	0.19845 (6)	0.93877 (9)	0.24696 (3)	0.05245 (18)	
S3	0.25794 (7)	0.52236 (9)	0.23740 (3)	0.05581 (19)	
O4	0.27371 (14)	0.7395 (2)	0.50559 (7)	0.0489 (4)	
O5	0.48199 (17)	0.7569 (3)	0.47037 (9)	0.0673 (5)	
N6	0.37617 (18)	0.7922 (3)	0.17126 (9)	0.0522 (5)	
C7	−0.0832 (2)	0.7267 (3)	0.53965 (11)	0.0416 (5)	
C8	−0.1507 (2)	0.7599 (3)	0.47806 (12)	0.0442 (5)	
H8	−0.2465	0.7639	0.4730	0.053*	
C9	−0.0740 (2)	0.7870 (3)	0.42398 (11)	0.0413 (5)	
H9	−0.1192	0.8099	0.3823	0.050*	
C10	0.07040 (19)	0.7808 (3)	0.43027 (10)	0.0349 (4)	
C11	0.1331 (2)	0.7472 (3)	0.49375 (10)	0.0369 (4)	
C12	0.0577 (2)	0.7200 (3)	0.54875 (11)	0.0442 (5)	
H12	0.1017	0.6977	0.5908	0.053*	
C13	0.15923 (19)	0.8083 (3)	0.37605 (10)	0.0368 (4)	
C14	0.2966 (2)	0.8017 (3)	0.38965 (11)	0.0419 (5)	
H14	0.3529	0.8210	0.3549	0.050*	
C15	0.3606 (2)	0.7662 (3)	0.45538 (11)	0.0455 (5)	
C16	0.0936 (2)	0.8373 (4)	0.30683 (11)	0.0501 (6)	
H16A	0.0133	0.9169	0.3099	0.060*	
H16B	0.0609	0.7164	0.2898	0.060*	
C17	0.2867 (2)	0.7429 (3)	0.21486 (10)	0.0414 (5)	
C18	0.4622 (3)	0.6497 (5)	0.14199 (14)	0.0774 (9)	
H18A	0.5282	0.6018	0.1758	0.116*	
H18B	0.5098	0.7053	0.1068	0.116*	
H18C	0.4050	0.5488	0.1243	0.116*	
C19	0.4015 (3)	0.9852 (5)	0.15168 (14)	0.0722 (8)	
H19A	0.3167	1.0548	0.1500	0.108*	
H19B	0.4372	0.9864	0.1084	0.108*	
H19C	0.4673	1.0418	0.1837	0.108*	

### Atomic displacement parameters (Å^2^)

**Table:**

	*U*^11^	*U*^22^	*U*^33^	*U*^12^	*U*^13^	*U*^23^
Cl1	0.0719 (4)	0.0596 (4)	0.0660 (4)	0.0019 (3)	0.0351 (3)	−0.0003 (3)
S2	0.0520 (4)	0.0574 (4)	0.0487 (3)	0.0071 (3)	0.0093 (2)	0.0123 (3)
S3	0.0611 (4)	0.0543 (4)	0.0515 (4)	−0.0088 (3)	0.0010 (3)	0.0002 (3)
O4	0.0347 (8)	0.0685 (11)	0.0424 (8)	0.0031 (7)	−0.0036 (6)	−0.0018 (7)
O5	0.0337 (9)	0.1053 (16)	0.0615 (11)	0.0061 (9)	−0.0057 (8)	−0.0083 (10)
N6	0.0336 (10)	0.0785 (15)	0.0446 (10)	0.0000 (9)	0.0038 (8)	0.0131 (10)
C7	0.0477 (12)	0.0289 (10)	0.0503 (12)	−0.0002 (8)	0.0180 (9)	−0.0030 (8)
C8	0.0338 (11)	0.0402 (12)	0.0597 (13)	0.0004 (8)	0.0101 (9)	−0.0016 (9)
C9	0.0339 (10)	0.0405 (12)	0.0492 (12)	0.0014 (8)	0.0012 (8)	0.0022 (9)
C10	0.0329 (10)	0.0290 (9)	0.0428 (10)	0.0004 (7)	0.0030 (8)	−0.0025 (8)
C11	0.0337 (10)	0.0329 (10)	0.0439 (11)	0.0015 (7)	0.0029 (8)	−0.0039 (8)
C12	0.0517 (13)	0.0398 (12)	0.0414 (11)	0.0030 (9)	0.0053 (9)	−0.0024 (9)
C13	0.0317 (10)	0.0375 (11)	0.0410 (10)	0.0018 (8)	0.0016 (8)	−0.0001 (8)
C14	0.0342 (10)	0.0485 (12)	0.0432 (11)	−0.0004 (9)	0.0040 (8)	−0.0017 (9)
C15	0.0340 (11)	0.0521 (13)	0.0503 (12)	0.0019 (9)	0.0017 (9)	−0.0057 (10)
C16	0.0333 (11)	0.0751 (17)	0.0423 (12)	0.0060 (10)	0.0052 (9)	0.0067 (11)
C17	0.0291 (10)	0.0612 (14)	0.0328 (10)	−0.0022 (9)	−0.0047 (7)	0.0045 (9)
C18	0.0583 (17)	0.120 (3)	0.0564 (16)	0.0281 (17)	0.0192 (13)	0.0130 (16)
C19	0.0536 (16)	0.098 (2)	0.0651 (17)	−0.0159 (15)	0.0067 (13)	0.0305 (16)

### Geometric parameters (Å, º)

**Table:**

Cl1—C7	1.737 (2)	C10—C11	1.392 (3)
S2—C17	1.783 (2)	C10—C13	1.454 (3)
S2—C16	1.788 (2)	C11—C12	1.386 (3)
S3—C17	1.663 (2)	C12—H12	0.9300
O4—C11	1.371 (2)	C13—C14	1.343 (3)
O4—C15	1.378 (3)	C13—C16	1.499 (3)
O5—C15	1.198 (3)	C14—C15	1.438 (3)
N6—C17	1.329 (3)	C14—H14	0.9300
N6—C19	1.455 (4)	C16—H16A	0.9700
N6—C18	1.466 (3)	C16—H16B	0.9700
C7—C12	1.369 (3)	C18—H18A	0.9600
C7—C8	1.376 (3)	C18—H18B	0.9600
C8—C9	1.378 (3)	C18—H18C	0.9600
C8—H8	0.9300	C19—H19A	0.9600
C9—C10	1.401 (3)	C19—H19B	0.9600
C9—H9	0.9300	C19—H19C	0.9600
			
C17—S2—C16	104.05 (11)	C13—C14—H14	118.4
C11—O4—C15	121.80 (16)	C15—C14—H14	118.4
C17—N6—C19	124.1 (2)	O5—C15—O4	117.2 (2)
C17—N6—C18	120.3 (2)	O5—C15—C14	126.1 (2)
C19—N6—C18	115.5 (2)	O4—C15—C14	116.74 (18)
C12—C7—C8	121.98 (19)	C13—C16—S2	117.08 (16)
C12—C7—Cl1	117.93 (18)	C13—C16—H16A	108.0
C8—C7—Cl1	120.08 (17)	S2—C16—H16A	108.0
C7—C8—C9	118.91 (19)	C13—C16—H16B	108.0
C7—C8—H8	120.5	S2—C16—H16B	108.0
C9—C8—H8	120.5	H16A—C16—H16B	107.3
C8—C9—C10	121.6 (2)	N6—C17—S3	124.10 (19)
C8—C9—H9	119.2	N6—C17—S2	112.96 (17)
C10—C9—H9	119.2	S3—C17—S2	122.94 (12)
C11—C10—C9	116.96 (19)	N6—C18—H18A	109.5
C11—C10—C13	117.78 (17)	N6—C18—H18B	109.5
C9—C10—C13	125.25 (18)	H18A—C18—H18B	109.5
O4—C11—C12	115.97 (18)	N6—C18—H18C	109.5
O4—C11—C10	121.78 (18)	H18A—C18—H18C	109.5
C12—C11—C10	122.25 (19)	H18B—C18—H18C	109.5
C7—C12—C11	118.3 (2)	N6—C19—H19A	109.5
C7—C12—H12	120.9	N6—C19—H19B	109.5
C11—C12—H12	120.9	H19A—C19—H19B	109.5
C14—C13—C10	118.78 (18)	N6—C19—H19C	109.5
C14—C13—C16	122.65 (18)	H19A—C19—H19C	109.5
C10—C13—C16	118.55 (17)	H19B—C19—H19C	109.5
C13—C14—C15	123.11 (19)		
			
C12—C7—C8—C9	0.0 (3)	C11—C10—C13—C16	177.7 (2)
Cl1—C7—C8—C9	−179.30 (16)	C9—C10—C13—C16	−2.9 (3)
C7—C8—C9—C10	0.3 (3)	C10—C13—C14—C15	0.9 (3)
C8—C9—C10—C11	−0.4 (3)	C16—C13—C14—C15	−177.3 (2)
C8—C9—C10—C13	−179.84 (19)	C11—O4—C15—O5	−180.0 (2)
C15—O4—C11—C12	−179.28 (19)	C11—O4—C15—C14	−0.3 (3)
C15—O4—C11—C10	0.5 (3)	C13—C14—C15—O5	179.2 (2)
C9—C10—C11—O4	−179.57 (18)	C13—C14—C15—O4	−0.4 (3)
C13—C10—C11—O4	−0.1 (3)	C14—C13—C16—S2	−20.1 (3)
C9—C10—C11—C12	0.2 (3)	C10—C13—C16—S2	161.75 (16)
C13—C10—C11—C12	179.74 (18)	C17—S2—C16—C13	85.7 (2)
C8—C7—C12—C11	−0.1 (3)	C19—N6—C17—S3	−179.84 (18)
Cl1—C7—C12—C11	179.17 (15)	C18—N6—C17—S3	−3.0 (3)
O4—C11—C12—C7	179.83 (18)	C19—N6—C17—S2	0.5 (3)
C10—C11—C12—C7	0.0 (3)	C18—N6—C17—S2	177.35 (18)
C11—C10—C13—C14	−0.6 (3)	C16—S2—C17—N6	−176.86 (15)
C9—C10—C13—C14	178.8 (2)	C16—S2—C17—S3	3.46 (16)

### Hydrogen-bond geometry (Å, º)

**Table:**

*D*—H···*A*	*D*—H	H···*A*	*D*···*A*	*D*—H···*A*
C16—H16*A*···S3^i^	0.97	2.84	3.707 (2)	150

Symmetry code: (i) −*x*, *y*+1/2, −*z*+1/2.
