# Supplementary figures and images for: Crystal structure of (7-chloro-2-oxo-2H-chromen-4-yl)methyl N,N-di­methyl­carbamodi­thio­ate
Source: Acta Crystallogr E Crystallogr Commun. 2015 Mar 28;71(Pt 4):o263–4. doi: 10.1107/S2056989015005678 (PMC4438814; doi:10.1107/S2056989015005678)

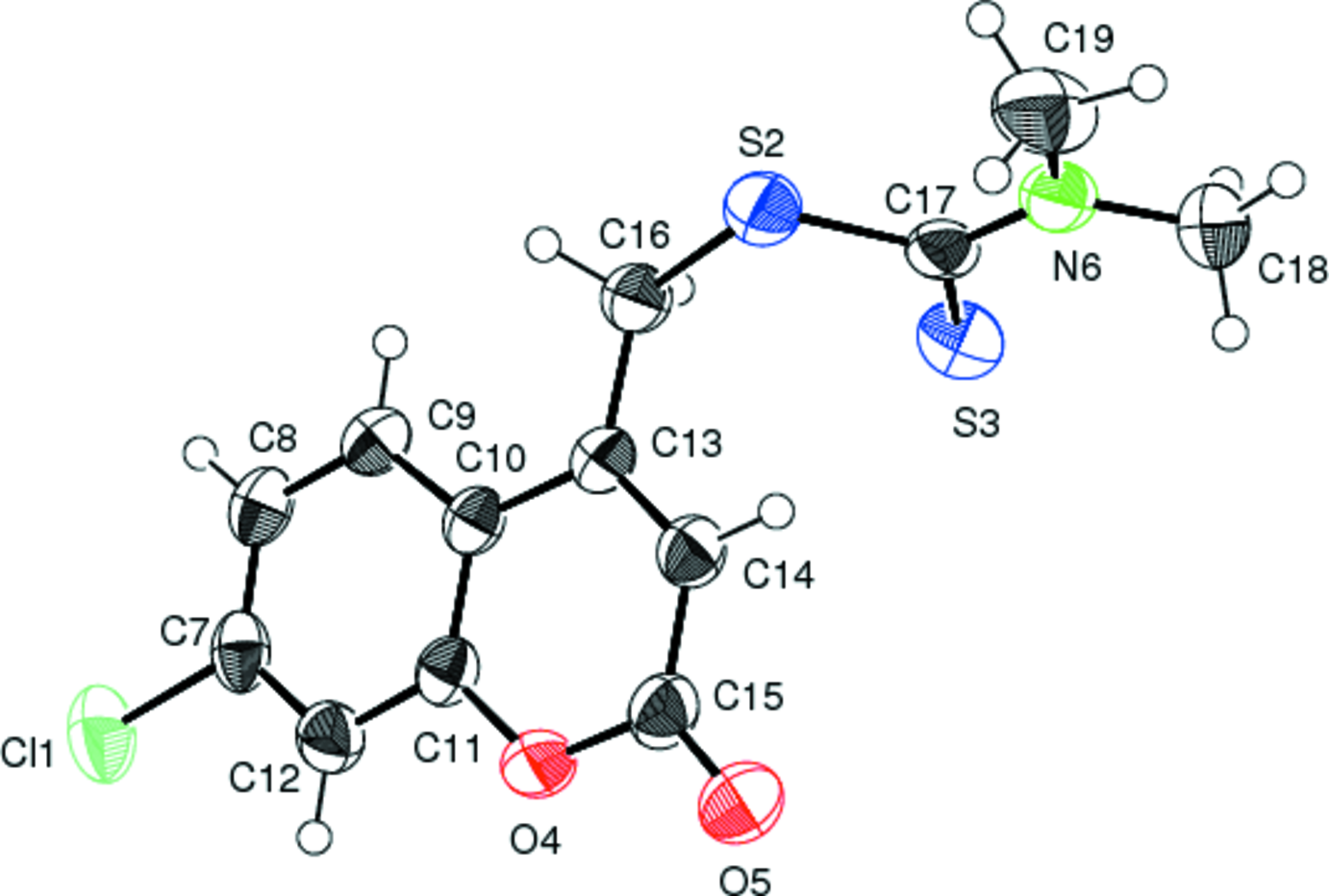

Supplement: Supplementary file 4 [file e-71-0o263-fig1.tif]

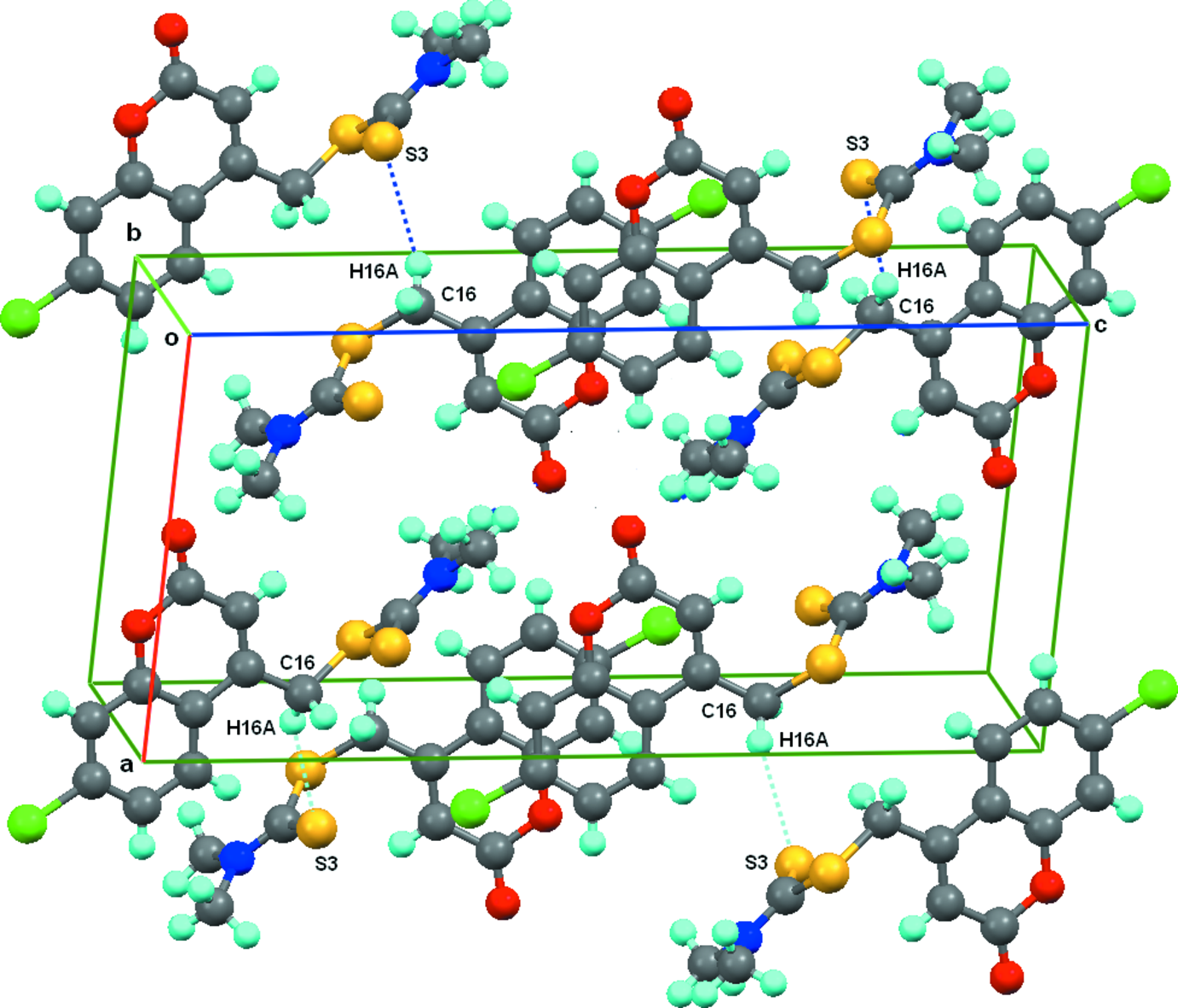

Supplement: Supplementary file 5 [file e-71-0o263-fig2.tif]
